# Coherent light-emitting metasurfaces based on bound states in the continuum

**DOI:** 10.1515/nanoph-2024-0040

**Published:** 2024-04-22

**Authors:** Soheil Farazi, Srinivas Tadigadapa

**Affiliations:** Department of Electrical and Computer Engineering, 1848 Northeastern University, Boston, MA 02115, USA

**Keywords:** bound states in the continuum, metasurfaces, thermal emission, light sources, Friedrich-Wintgen interferences, light-matter interaction

## Abstract

An emergent need exists for solid state tunable coherent light emitters in the mid-infrared range for spectroscopy, sensing, and communication applications where current light sources are dominated by spontaneous emitters. This paper demonstrates a distinct class of coherent thermal emitters operating in the mid-infrared wavelength regime. The structure of the light source consists of a dielectric metasurface fabricated on a phononic substrate. In this study, we present the first implementation of off-Γ Friedrich–Wintgen bound states in the continuum at mid-infrared wavelengths suitable for developing the next generation of coherent light emitters. Numerical analysis of the emissivity spectrum reveals the interference of resonances leading to avoided crossings and the formation of Friedrich–Wintgen bound states in the radiation spectrum. Additionally, significant localized field enhancements are observed within the metasurface at operating wavelengths. The emissivity spectra measured by reflectivity and emission experiments exhibit temporally coherent emission peaks in the vicinity of the bound state in the continuum, the first such demonstration in the mid-infrared region for wavelengths longer than 7 µm. These results represent a new approach for significant advancement in realizing mid-infrared coherent light emitters with promising implications for future technologies.

## 1 Introduction

The mid-infrared (mid-IR) wavelength spectrum is one of the most abundantly available forms of radiation due to the black body emission peak at room temperature. It is also a range of wavelengths that can be used to study and identify molecular species with their unique vibrational and rotational spectral signatures. Moreover, it is rapidly emerging as a critical wavelength band for terahertz (THz) and optical communication systems. The mid-IR range is dominated by thermal emitters, which are spontaneous, incoherent, and broadband in nature. However, the emergent applications in mid-IR such as lasing [1], [2], [3], [4], sensing [5], [6], [7], and optical communication [8], [9] rely on coherent light emitters. The considerable size and cost of the current IR spectrometers are primarily due to the large size of the IR emitters and detectors. Therefore, the development of chip-scale and low-cost mid-IR sources will likely be of great importance for portable spectrometers as well [10], [11].

As a result of the available wide bandgap semiconductors that are capable of emitting light at room temperature, narrowband emitters have been developed with considerable success in the visible [12], [13], [14] and near-infrared (NIR) [15], [16], [17] wavelength spectra. However, manufacturing narrowband light emitters in the mid-IR range, particularly for wavelengths longer than 7 µm, has remained challenging due to technological challenges and material limitations. For instance, the only semiconductor lasers operating at wavelengths longer than 7 µm are quantum cascade lasers (QCLs), which have complex structures. The manufacturing of these lasers requires specialized techniques, such as molecular beam epitaxy (MBE) and metal-organic chemical vapor deposition (MOCVD), as well as precision control of the thickness of multiple semiconductor layers to achieve the desired performance. Therefore, chip-scale coherent light emitters that are inexpensive, robust, and easy to realize are highly desirable.

Electromagnetic radiation can be controlled or manipulated using phononic and plasmonic materials [18], [19], [20], [21], [22]. Furthermore, these materials with surface phononic or plasmonic resonances can generate high-intensity radiation localized to the surface under thermal excitation due to the enhanced local density of photonic states. It has been demonstrated that when tailored periodic patterns are inscribed on such a material surface, the radiation out-couples to free space in the form of coherent mid-IR light beams [23]. However, the sharpness and the quality factor (Q-factor) of the emission peaks remain limited due to the intrinsic loss of the phononic and plasmonic materials [24], [25], [26].

This paper uses bound states in the continuum (BICs) to design and fabricate temporally coherent light emitters in the mid-IR range between 10 µm and 12 µm wavelengths. Bound states in the continuum are localized states, referred to as embedded eigenvalues of the system, that lie inside the radiation spectrum while they are confined with no radiation or leakage. In 1929, von Neumann and Wigner proposed the BICs in quantum mechanics [27]. Considering their nature as wave phenomena, BICs have been introduced to electromagnetics and optics and have become increasingly popular in recent years. In optics, BICs were first theoretically demonstrated in 2008 [28], and experimentally observed in 2011 [29]. Since then, several groups have investigated the various properties of BICs to boost their potential for designing photonic devices as they can provide strong field enhancement needed in many applications including biosensing [30], [31], imaging [32], [33], and nonlinear optics [34], [35]. Besides the phononic and plasmonic materials discussed earlier, dielectric materials are also capable of controlling and manipulating electromagnetic waves through the formation of BICs [36], [37], [38].

In wave systems like photonic platforms with periodic patterns, BICs are realized in a medium that is at least extended to infinity in one dimension [39]. Generally, BICs are categorized into two main groups. The first group is BICs localized due to the symmetry of the structure [40], [41], [42], [43], [44]. As soon as the symmetry of the system is broken, they start to radiate and become quasi-BICs (Q-BICs) that can be detected in the continuum spectrum. These types of BICs are called “symmetry-protected” or “Γ point” BICs trapped at Γ point of the dispersion diagram of the photonic structures. The second group is those BICs that stay localized even when the symmetry of the lattice is already broken. These are called “off-Γ” BICs and can be formed due to different mechanisms such as the interaction of coupled resonances, referred to as “Friedrich–Wintgen” BICs [45], [46], or by parameter tuning in the presence of a single resonance called “accidental” BICs [47].

In our study, we present the first implementation of an off-Γ Friedrich–Wintgen BIC at mid-IR wavelengths between 10 µm and 12 µm to achieve temporally coherent light emitters. This goal is accomplished by stimulating Friedrich-Wintgen coupled resonances within a dielectric metasurface fabricated on a phononic substrate. Our experimental results provide compelling evidence of Friedrich-Wintgen resonances at the wavelengths of interest. Additionally, we observed temporally coherent emission peaks in the near-BIC regime. As compared to the broadband radiation generated in platforms supporting Γ point and symmetry-protected BICs [48], our work based on off-Γ Friedrich–Wintgen BICs enables temporally coherent emission in the mid-IR range.

## 2 Results and discussion

When two Friedrich–Wintgen resonances pass each other as a function of a continuous parameter, such as wavelength or incidence angle, an avoided crossing occurs due to resonance interference. At a certain continuous parameter, one of the resonances vanishes entirely and hence becomes a BIC, resulting in the detection of a single resonance at the BIC point. This type of BIC was first established by Friedrich and Wingten [45]. By applying temporal coupled-mode theory [39], [49], [50], [51], Friedrich–Wintgen BICs can be better understood. For two resonance states interfering in the same resonator with resonance frequencies *ω*_1,2_, the system can be represented by the effective Hamiltonian

(1) $$\tilde{H}=\left(\begin{array}{cc} \omega_{1}-i\gamma_{1} & \kappa -i\sqrt{\gamma_{1}\gamma_{2}}\\ \kappa -i\sqrt{\gamma_{1}\gamma_{2}} & \omega_{2}-i\gamma_{2} \end{array}\right)$$

where *γ*_1,2_ are the linewidths of the resonances and *κ* is the near-field coupling constant. A Friedrich–Wintgen BIC is formed and decoupled from the continuum when one of the eigenvalues becomes purely real. Accordingly, it can be shown that for the condition [45], [52]

(2) $$\kappa \left(\gamma_{1}-\gamma_{2}\right)=\sqrt{\gamma_{1}\gamma_{2}}\left(\omega_{1}-\omega_{2}\right)$$

one of the eigenvalues has an imaginary part of zero, Im(*σ*_+_) = 0, and turns into a BIC with a zero linewidth, while for the other one Im(*σ*_−_) = −(*γ*_1_ + *γ*_2_).

**Figure 1:**
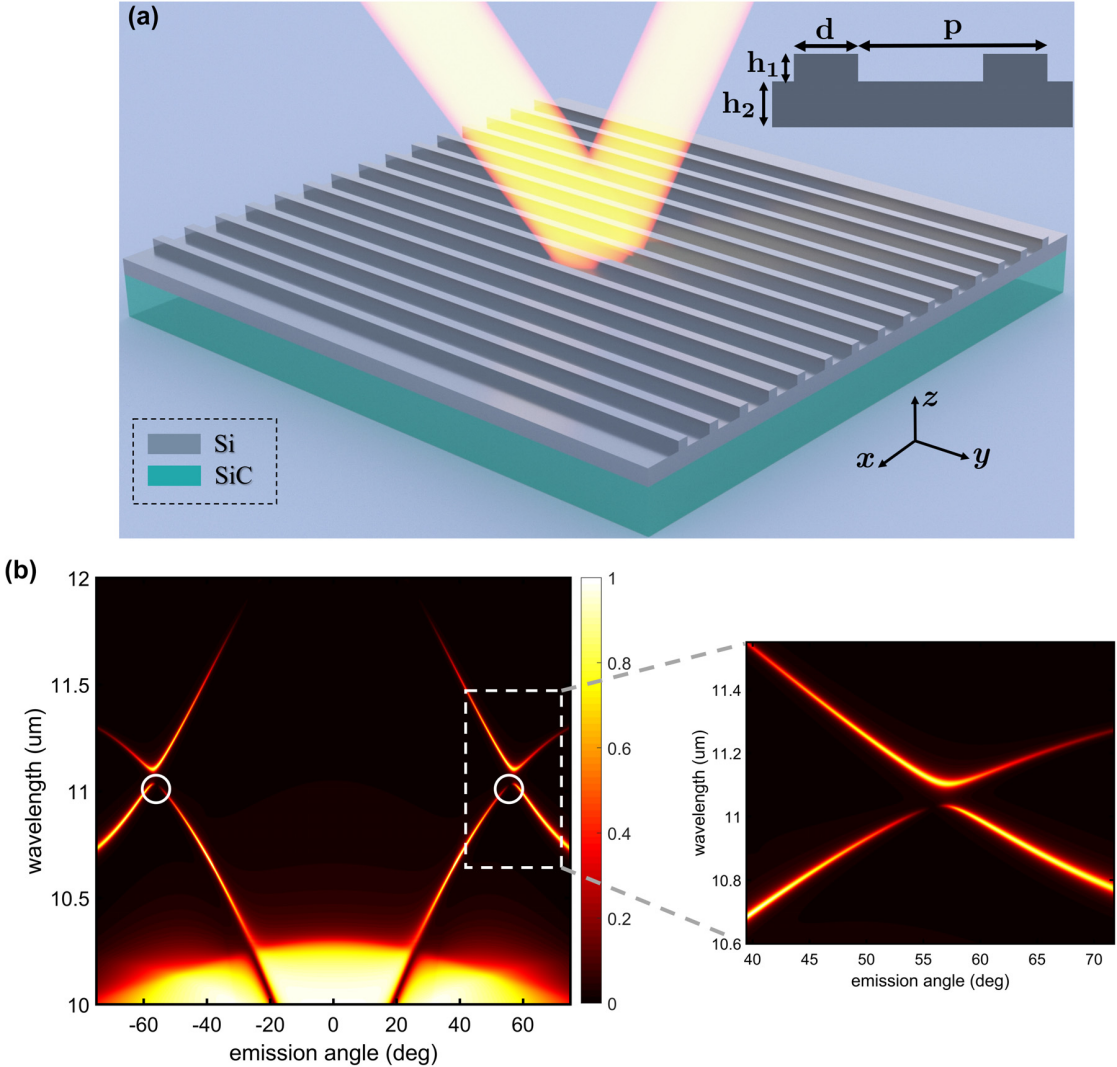
Coherent light-emitting metasurface. (a) Schematic of the phononic-based metasurface including SiC substrate (teal) and silicon film and grating patterns (grey) with the period of the patterns *p* = 6.6 µm, width *d* = 2 µm, grating depth *h*_1_ = 0.5 µm, and film thickness *h*_2_ = 1.2 µm. (b) Emissivity as a function of emission angle and wavelength. White circles indicate BIC points, and the white dashed box shows the near-BIC regime. The right panel in (b) illustrates an expanded view of the white dashed box.

We have utilized Friedrich–Wingten BICs to realize coherent light emitters. The geometry of the proposed light-emitting metasurface is illustrated in Figure 1(a) where *p* = 6.6 µm, *d* = 2 µm, *h*_1_ = 0.5 µm, and *h*_2_ = 1.2 µm. The substrate is silicon carbide (SiC) which is a phononic material with a negative real part for permittivity in the Reststrahlen band between wavelengths of 10.3 µm and 12.5 µm. In this range, the complex permittivity of SiC can be defined by an oscillator model [53], [54]

(3) $$\epsilon \left(\omega \right)=\epsilon_{\infty }\left(1+\frac{\omega_{L}^{2}-\omega_{T}^{2}}{\omega_{T}^{2}-\omega^{2}-i\tau \omega }\right)$$

with *ϵ*_∞_ = 6.7, *ω*_*L*_ = 969 cm^−1^, *ω*_*T*_ = 793 cm^−1^, and *τ* = 4.76 cm^−1^. The dielectric layer on top of the substrate is silicon with optical properties described in Ref. [55]. Figure 1(b) shows the emissivity as a function of emission angle and wavelength for the TE polarization (electric field is along the length of the nano bars) obtained by Kirchhoff’s law as 1 − *R* where *R* is the sum of reflection coefficients of all propagating diffraction orders of the system calculated by rigorous coupled-wave analysis (RCWA) method [22], [56]. The white circles in Figure 1(b) indicate the Friedrich–Wintgen BIC points where the linewidth of the resonances becomes zero. Figure 1(b) displays an expanded view of the white dashed box in the right panel. This box shows the near-BIC regime at which Friedrich–Wintgen BIC is evident. As the emission angle increases, the resonances approach one another until at one point, the linewidth of one of the resonances drops to zero due to destructive interference of the resonances, resulting in an avoided crossing for the two resonances and a band flip [45], [57], [58]. As a result of the near-field coupling through the silicon layer (*κ* ≠ 0), BICs will not occur precisely at the crossing point [50]. While the linewidth of the resonance becomes zero at the BIC point, emission peaks with high amplitude and Q-factor can be achieved in the near-BIC regime.

**Figure 2:**
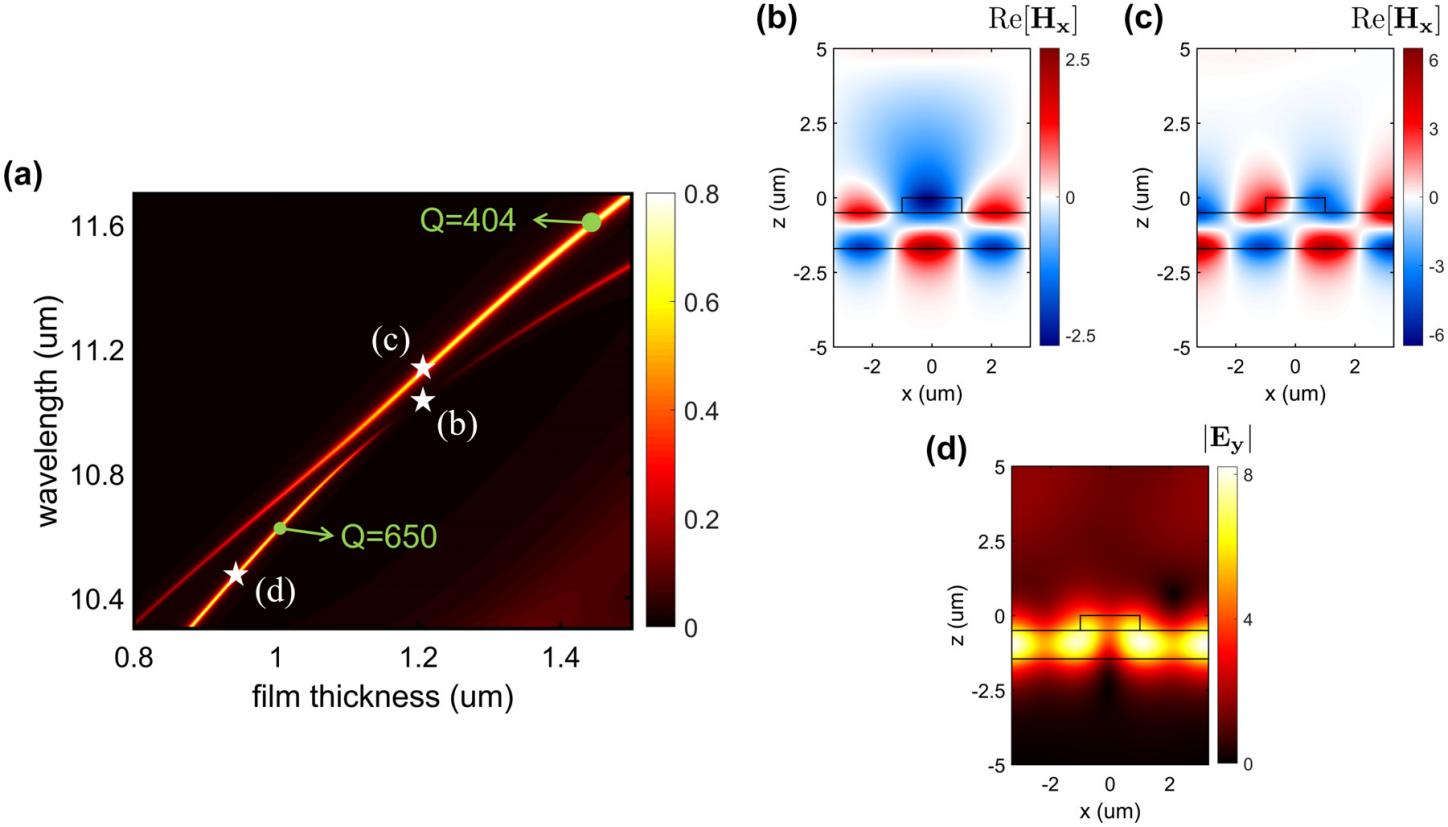
Numerical simulation of emissivity and field distribution. (a) Emissivity as a function of dielectric thickness, *h*_2_, and wavelength at emission angle *θ* = 56°. The green dots indicate the locations of emission peaks for which Q-factors have been calculated. Magnetic field distribution within the structure for dielectric thickness *h*_2_ = 1.2 μm at wavelength (b) *λ* = 11 µm, the BIC point and (c) *λ* = 11.12 µm, the near-BIC point. (d) Electric field profile at emission peak located at *λ* = 10.5 µm where *h*_2_ = 0.95 µm which demonstrates field confinement within the dielectric layer. In (a), stars indicate the locations of the field profiles illustrated in (b–d).

The effect of the dielectric layer, here the silicon film, as a function of its thickness, *h*_2_, and wavelength has been numerically investigated. Figure 2(a) illustrates the interaction between the resonances and the avoided crossing at an emission angle *θ* = 56°, corresponding to the angle at which the Friedrich–Wintgen BIC is formed in Figure 1(b). The effect of film thickness on the linewidth and location of both resonances is evident. As a result, the Q-factor of the emission peak can be engineered from 404 to 650 by modifying the thickness of the dielectric layer.

The nearfield distributions within the structure have also been studied. For the fixed dielectric thickness of *h*_2_ = 1.2 µm, Figure 2(b) and (c) depict the magnetic field profiles of the BIC point and the emission peak located at wavelengths *λ* = 11 µm, and *λ* = 11.12 µm, respectively, which are marked in Figure 2(a) with stars. Figure 2(c) clearly displays a significant enhancement of the magnetic field and light–matter interaction at the interfaces which are well-known characteristics of the near-BIC regime [57], [59]. Figure 2(d) also illustrates the distribution of the electric field for the emission peak at *λ* = 10.5 µm where *h*_2_ = 0.95 µm, demonstrating a strong field localization inside the dielectric layer at the emission peak.

**Figure 3:**
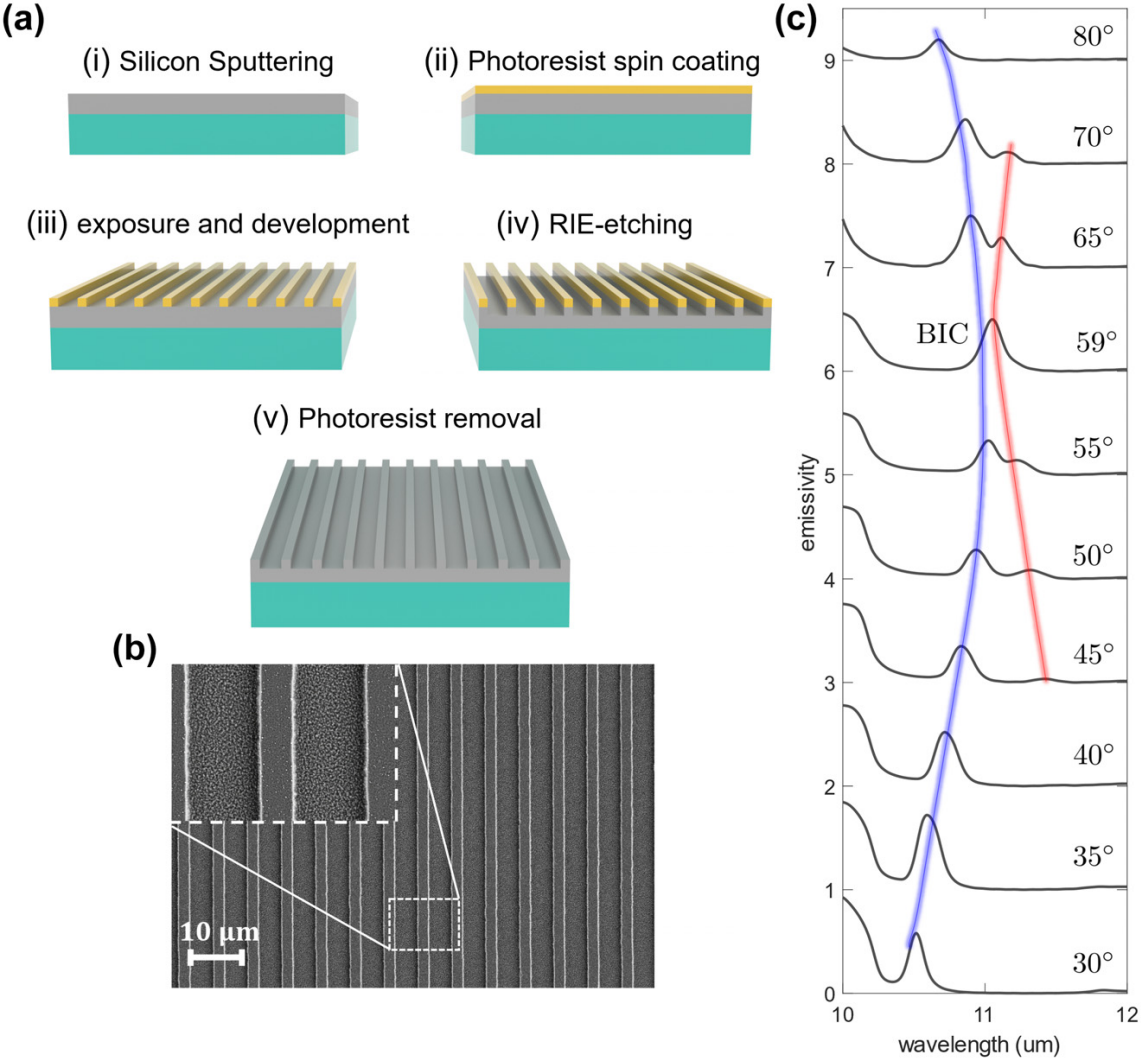
Fabrication process and reflectivity measurement. (a) Fabrication process of the light-emitting metasurface. (b) SEM image of the fabricated phononic-based metasurface where *p* = 6.6 µm, *d* = 2 µm, *h*_1_ = 0.5 µm, and *h*_2_ = 1.2 µm. The inset shows an expanded view of the grooves and nano bars. (c) Measured emissivity from the fabricated light-emitting metasurface as a function of wavelength at various angles demonstrating the Friedrich–Wintgen BIC formation. The blue and red lines in (c) indicate the trajectory of interference between Friedrich–Wintgen resonances.

Figure 3(a) presents the fabrication process of the light-emitting metasurface. To fabricate the metasurface, a silicon layer with 1.7 µm thickness was sputter deposited on a 4H–SiC substrate. Then, the silicon grating was patterned using a photolithography process followed by dry etching in an ICP-RIE tool. This process resulted in silicon bars of width 2 µm, with a pitch of 6.6 µm. Figure 3(b) displays an SEM image of the metasurface. After fabrication of the device, the emissivity of the light emitter was determined using a Bruker FTIR spectrometer model Invenio-R by acquiring reflectivity spectra in response to a TE-polarized incident light. The measured emissivity at different emission angles is depicted in Figure 3(c). It is evident that at emission angles below 45°, there is a single emission peak. However, at higher angles, a second emission peak emerges at longer wavelengths. Upon increasing the angle, the two emission peaks corresponding to Friedrich–Wintgen resonances move toward each other until the Friedrich–Wintgen BIC is formed, resulting in the detection of only one emission peak. Moreover, the measured emissivity spectra indicate a temporally coherent emission in the near-BIC regime. The temporal coherence length of the light source can be obtained by [13], [60]

(4) $$L_{c}=c\;\tau_{c}=\sqrt{\frac{2\;\ln2}{\pi }}\frac{\lambda^{2}}{\Delta \lambda }$$

where *τ*_*c*_ is the coherence time, *c* is the speed of light, *λ* and Δ*λ* are the central wavelength and the full width half maximum (FWHM) of the emission peak, respectively. At *θ* = 30°, the temporal coherence length of the emission peak located at *λ* = 10.52 µm is *L*_*c*_ = 0.62 mm which is as large as 59*λ*. The emission peaks exhibit a Q-factor of 85, which is significantly higher than experimental results obtained from Γ point BICs operating within this wavelength range (10 µm–12 µm) [48]. Due to impurities in the silicon film and optical losses associated with sputtered silicon, the experimental Q-factors are, however, lower than the predicted numerical values of the quasi-BIC emissions. While the imaginary part of the permittivity determining the optical loss of the system is negligible for single crystalline silicon [55], the amount of optical loss can be considerable for amorphous silicon (a-Si) depending upon the silicon structure, defects, and doping [61]. By improving the quality of the deposited silicon film the optical loss of the dielectric layer will be decreased, resulting in much higher Q-factor emission peaks.

**Figure 4:**
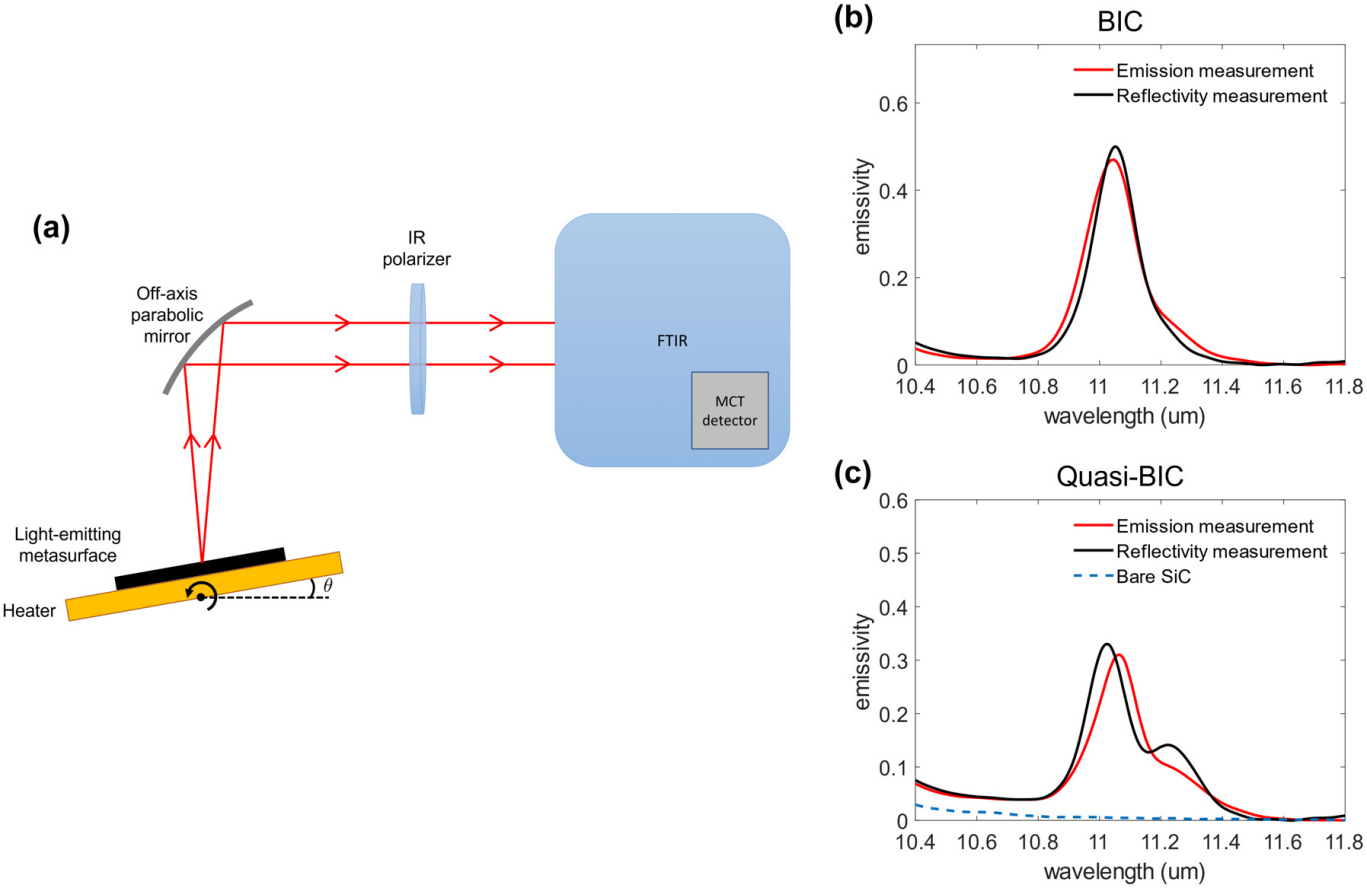
Emission measurement. (a) Simplified schematic of the experimental setup used for thermal emission measurements to collect the emitted light from the fabricated metasurface by an FTIR spectrometer. (b) The emissivity spectrum determined from the thermal emission experiment (red solid line) at emission angles (b) *θ* = 59° and (c) *θ* = 55°, corresponding to BIC and quasi-BIC radiation spectrum, respectively, which confirm the emissivity spectrum obtained from reflectivity measurement (black solid line). The blue dashed line in (c) illustrates the emissivity of the bare 4H–SiC substrate.

The emissivity spectrum can also be determined by measuring the thermal radiation emitted from the metasurface [62], [63]. Figure 4(a) displays a schematic representation of the experimental setup for determining emissivity at a fixed observation angle. During the measurement, the sample was heated to 96 °C in order to increase the intensity of the signal to be detected by a mercury–cadmium–telluride (MCT) detector. The emissivity spectrum can be quantified as

(5) $$\begin{array}{ll} \varepsilon \left(\lambda \right) & =\varepsilon_{b}\left(\lambda \right)\times \left[\frac{S_{e}\left(\lambda ,T_{e}\right)-S_{r}\left(\lambda ,T_{r}\right)}{S_{b}\left(\lambda ,T_{b}\right)-S_{r}\left(\lambda ,T_{r}\right)}\right]\\ & \;\times \left[\frac{B\left(\lambda ,T_{b}\right)-B\left(\lambda ,T_{r}\right)}{B\left(\lambda ,T_{e}\right)-B\left(\lambda ,T_{r}\right)}\right] \end{array}$$

where *ɛ*_*b*_ is the known emissivity spectrum of a blackbody reference source. *S*_*e*_, *S*_*b*_, and *S*_*r*_ represent the intensity measured from the light-emitting metasurface, blackbody source, and background room radiation, respectively, at the temperature *T*_*e*_, *T*_*b*_, and *T*_*r*_. The Plank function at temperature *T* corresponding to the blackbody radiation distribution is given by

(6) $$B\left(\lambda ,T\right)=\frac{2hc^{2}}{\lambda^{5}}\frac{1}{e^{hc/\lambda k_{B}T}-1}$$

where *h* and *k*_*B*_ are the Planck and Boltzmann constants, respectively. Figure 4(b) shows the TE-polarized emission from the metasurface as determined by thermal emission measurements taken at fixed observation angle 59° which corresponds to the formation of the Friedrich–Wintgen BIC. Figure 4(c) also displays emissivity at angle 55°, demonstrating the quasi-BIC radiation spectrum. In terms of emissivity, there is a very consistent agreement between thermal emission and reflectivity measurements. The emission peak determined by thermal radiation experiments, however, appears broader than the reflectivity measurement result due to the imaginary part of SiC permittivity increasing slightly with temperature [62]. The total emitted power per unit area can be obtained by

(7) $$P=\overset{\lambda_{2}}{\underset{\lambda_{1}}{\int }}\varepsilon \left(\lambda \right)\cdot B\left(\lambda ,T\right)\;d\lambda .$$

For the single coherent emission peak at *θ* = 59°, illustrated in Figure 4(b), the total emitted power per unit area is 2.52 W m^−2^. Considering our chip size, 20 mm by 20 mm, the coherent light-emitting metasurface is capable of emitting 1 mW, indicating that the light emitter has sufficient brightness for medical applications [64]. This amount of power is 11 times greater than the total power emitted by a bare SiC substrate at the wavelengths of interest, and it can be further amplified by increasing the temperature.

In addition to the findings presented, it is pertinent to discuss the aspect of unwanted power loss at certain emission angles, specifically at *θ* = 59°. At this angle, our numerical analysis indicates an unwanted power loss of 25 %, attributed primarily to first-order diffraction into the substrate. This phenomenon has a noticeable impact on the total radiated power, potentially affecting the efficiency of the coherent light-emitting metasurface. Despite this, it is crucial to highlight that the coherent light emitter maintains a substantial output, capable of providing 1 mW of power at the desired emission angle of *θ* = 59°. This level of performance, even in the face of such power loss, underscores the suitability of the emitter for practical applications as previously mentioned. It is also important to note that the observed unwanted power loss is not a fixed limitation but rather a parameter dependent on the emission angle. By adjusting to lower emission angles, the unwanted power loss associated with first-order diffraction can be minimized or even eliminated for emission angles less than *θ* = 36°. This adaptability offers a pathway to optimize the configuration of the metasurface for enhanced performance, allowing for a balance between desired emission characteristics and minimal energy loss.

In order to estimate the absorption losses linewidth associated with fabrication imperfection, the measured emission peak at the emission angle *θ* = 59° can be represented by a Fano formula given by [65], [66]

(8) $$F\left(\omega \right)=\frac{2D}{1+x^{2}}.$$

Here, *D* is a coefficient corresponding to the peak amplitude, *x* = (*ω* − *ω*_0_)/*γ*_0_ is the normalized frequency offset where *ω*_0_ and *γ*_0_ are the resonance frequency and broadening, respectively. According to the Fano formula, the measured emission peak has a linewidth of Δ*ω*_exp_ = 12.2 cm^−1^. By considering the linewidth of Δ*ω*_*n*_ = 2 cm^−1^ calculated from the numerical result, the absorption losses linewidth associated with material optical losses and fabrication imperfections is estimated to be 10.2 cm^−1^. In the pursuit of advancing metasurface designs within the realms of fabrication and material limitations, particularly those influencing the amplitude and Q-factor of emission peaks in the near-BIC regime, the concept of supercritical coupling emerges as a compelling strategy [67]. This approach, closely aligned with the Friedrich–Wintgen condition, presents a viable pathway to increase emissivity amplitude by leveraging the unique interplay between radiative and non-radiative losses to enhance field intensities beyond conventional limits. Incorporating this method could boost emissivity, offering a prospective solution to the challenges posed by current fabrication and material constraints.

## 3 Conclusions

In this study, we demonstrated the first implementation of off-Γ Friedrich–Wintgen BICs in the mid-IR range. Through the formation of BICs, we have developed a temporally coherent phononic-based light-emitting metasurface at mid-IR wavelengths. In spite of the realistic intrinsic loss of the phononic substrate, the demonstrated thermal source is capable of producing high Q-factor emission peaks. As a result of the interaction of two resonances, Friedrich–Wintgen BICs were observed, which led to the avoidance of resonance crossings. The effect of the dielectric layer on both resonances has been investigated. The near BIC field distribution also exhibits significant enhancement and localization within the dielectric layer. A reflectivity measurement was conducted after the metasurface had been fabricated to assess the emissivity spectrum of the emitter. The experimental results of reflectivity revealed quasi-BIC emissions and interference arising from Friedrich–Wintgen resonances. In order to measure the emitted light directly from the metasurface, a thermal emission experiment was performed that confirmed the reflectivity results. According to both experimental results, the emission peak was temporally coherent. In this first report of the mid-IR emitter based on off-Γ Friedrich–Wintgen BICs, the peak amplitude and Q-factor are less than the numerical values due to optical losses associated with the sputtered dielectric film. A much higher Q-factor can be achieved by improving the quality of the deposited dielectric layer. Light emitters such as the one demonstrated here could be used for advancing mid-IR spectroscopy, optical communications, and photonic integrated circuits, thereby paving the way for the development of next generation optical devices in the mid-IR range. Their cost-effectiveness, simple fabrication process, and high spectral purity enable the realization of miniaturized spectrometers, enhance data transmission rates in optical communications, and facilitate the integration of coherent light sources into photonic chips.

## 4 Methods

### 4.1 Numerical simulations

In our study, the numerical analyses of emissivity and near-field distributions were conducted using the RCWA method [22], tailored to the specific requirements of metasurface modeling. This approach enabled the precise calculation of reflectivity across various diffracted orders, from which emissivity was derived as *ɛ* = 1 − *R* where *R* is the sum of reflection coefficients of all propagating diffraction orders of the system. The analysis accounted for incident light characteristics corresponding to our experimental conditions, ensuring a rigorous and comprehensive modeling of the optical response of the metasurface. The iterative inclusion of a sufficient number of Fourier harmonics guaranteed the convergence of the calculations, ensuring the reliability of our numerical results.

### 4.2 Experimental measurements

#### 4.2.1 Reflectivity measurement

In the present study, the reflectivity spectrum in response to a TE-polarized incident light was collected using a Bruker FTIR spectrometer model Invenio-R. The emissivity spectrum was determined according to Kirchhoff’s law as *ɛ*(*λ*, *θ*) = *A*(*λ*, *θ*) = 1 − *R*(*λ*, *θ*) where *A*(*λ*, *θ*) and *R*(*λ*, *θ*) represent absorptivity and reflectivity spectra, respectively.

#### 4.2.2 Thermal emission measurement

In order to determine the emissivity spectrum using the thermal radiation experiment, the light-emitting metasurface was mounted on an on-axis rotary stage to measure the emitted light at the desired observation angle. The metasurface was heated to 96 °C (the sample temperature was monitored by a thermometer) using a substrate heater to increase the intensity of the measured signal. An off-axis parabolic mirror was used to collimate the light emitted from the sample located at its focal point. The emitted light was collected by the mercury–cadmium–telluride (MCT) detector of the FTIR spectrometer after propagating through an IR polarizer and becoming TE-polarized. To obtain the emissivity by Eq. (5), the background radiation was collected through the input window of the FTIR spectrometer. Additionally, the signal from a blackbody reference source with emissivity *ɛ* = 0.8 operating at the temperature of 450 °C was also collected.
